# Risk factors and outcomes of prolonged recovery from delayed graft function after deceased kidney transplantation

**DOI:** 10.1080/0886022X.2020.1803084

**Published:** 2020-08-10

**Authors:** Huanxi Zhang, Qian Fu, Jinqi Liu, Jun Li, Ronghai Deng, Chenglin Wu, Weijian Nie, Xutao Chen, Longshan Liu, Changxi Wang

**Affiliations:** a Organ Transplant Center, The First Affiliated Hospital, Sun Yat-sen University, Guangzhou, China; b Zhongshan School of Medicine, Sun Yat-sen University, Guangzhou, China; c Guangdong Provincial Key Laboratory on Organ Donation and Transplant Immunology, Guangzhou, China; d Guangdong Provincial International Cooperation Base of Science and Technology (Organ Transplantation), Guangzhou, China

**Keywords:** Kidney transplantation, delayed graft function, acute kidney injury, graft survival

## Abstract

**Objective:**

We aimed to evaluate the effect of prolonged recovery from DGF on outcomes, using a new definition of DGF recovery time, among deceased donor kidney transplant recipients with DGF, and to examine the risk factors for prolonged recovery.

**Methods:**

From 2007 to 2016, 91 deceased donor kidney transplant recipients with DGF were retrospectively analyzed. DGF recovery time was defined as the time from transplantation to achieve a stable estimated glomerular filtration rate (eGFR). Recipients with a DGF recovery time greater than or equal to the median were assigned to the prolonged recovery group, while the others were assigned to the rapid recovery group.

**Result:**

The median DGF recovery time was 27 days. Donor terminal eGFR was significantly lower in the prolonged recovery group (*n* = 46) compared with the rapid recovery group (*n* = 45) (median 24.9 vs. 65.4 ml/min/1.73m^2^, *p* = 0.004). The eGFR at 1 year post-transplant in the prolonged recovery group was significantly lower than that in the rapid recovery group (50.6 ± 20.0 vs. 63.5 ± 21.4 ml/min/1.73m^2^, *p* = 0.005). The risk of adverse outcomes (acute rejection, pneumonia, graft failure, and death) was significantly greater in the prolonged recovery group (hazard ratio 2.604, 95% confidence interval 1.102–6.150, *p* = 0.029) compared with the rapid recovery group.

**Conclusion:**

Decreased donor terminal eGFR is a risk factor for prolonged recovery from DGF after deceased kidney transplantation. Prolonged DGF recovery time is associated with reduced graft function at 1-year post-transplant, and poor transplant outcome.

## Introduction

Kidney transplantation has become a routine procedure for the treatment of irreversible kidney failure [1]. Delayed graft function (DGF) is an early complication after kidney transplantation [2]. The reported incidence of DGF in recipients of kidneys from deceased donors has increased over the past decades [3]. The incidence of DGF is expected to rise further due to the use of expanded criteria donors (ECDs), and donation after cardiac death (DCD) which is associated with a higher rate of DGF than donation after brain death (DBD) [4–6]. However, the relation between long-term graft survival and DGF is unclear despite many studies examining this issue [7–13].

It is reasonable to infer that DGF has various subtypes based on cause, predisposing factors, and underlying mechanisms, and thus with differing prognoses [14,15]. In addition, the time required for graft function recovery after DGF varies greatly, ranging from primary non-function to the need for only 1 session of dialysis. DGF is the result of various injuries to the allograft (e.g. preexisting lesions, donor acute kidney injury, surgery-related injury, ischemia-reperfusion injury), while DGF recovery time reflects the balance between the severity of the injury and the repair capacity of the recipient. Therefore, DGF recovery time is considered a useful index to further categorize DGF, and may be more relevant to the prognosis. Studies have reported that prolonged DGF duration is associated with poorer allograft function [16–21], graft survival [17,22,23], and patient survival (infectious mortality) [16,17]. On the other hand, few studies have examined factors that predict the duration of DGF in cadaveric kidney transplantation [24].

**Figure 1.**
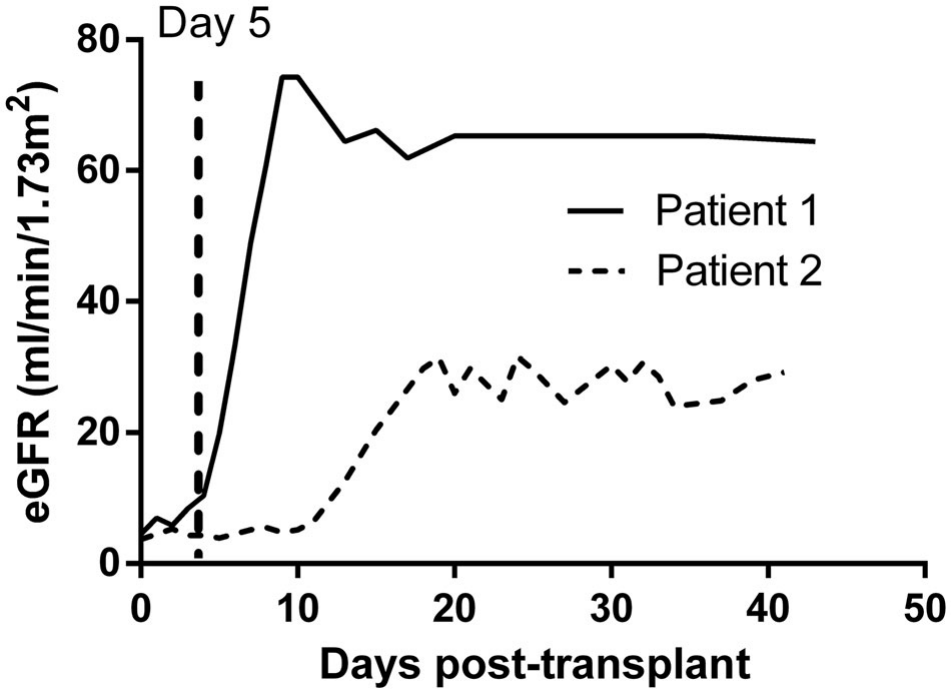
Two patterns of graft function recovery from delayed graft function. The two curves are drawn based on real data. Both patients stopped dialysis at Day 5 after kidney transplantation (TND = 5 days). However, it took patient 2 longer to reach a stable level of eGFR and a decrease in stable eGFR was also seen in patient 2. eGFR, estimated glomerular filtration rate; TND, time needed on dialysis after kidney transplantation.

Most studies define DGF duration as the time needed on dialysis (TND) after transplantation [17,18,20,21,23,24]. However, when investigating the pattern of DGF recovery after kidney transplantation, we found that patients with the same TND recovered at different speeds (Figure 1). We thus hypothesized that the time required to achieve stable allograft function, measured from the day of transplantation, may be more relevant to the prognosis than TND.

Thus, the purpose of this study was to evaluate the effect of prolonged recovery from DGF on outcomes, using a new definition of DGF recovery time, among deceased donor kidney transplant recipients with DGF, and to examine the risk factors for prolonged recovery.

## Methods

### Study population

The medical records of consecutive patients who received solitary renal transplantation from deceased donors [25] at the First Affiliated Hospital, Sun Yat-sen University between February 2007 and August 2016 were retrospectively reviewed. DGF was defined as requiring dialysis within the first week post-transplantation. DGF recovery time was defined as the time required to achieve stable allograft function (estimated glomerular filtration rate [GFR] did not change by more than 10% in the following week), as measured from the day of transplantation. DGF recovery time ≥ the median time was categorized as prolonged recovery, and DGF recovery time < the median time was categorized as rapid recovery. Patients were categorized into 1 of the 2 groups.

### Immunosuppression

For immunosuppression induction, patients received either anti-thymocyte globulin (ATG) or basiliximab. ATG was given at a dose of 50 mg during the transplant operation, and then daily for the next 2 days. For basiliximab, 20 mg was given on the day of surgery, and then again on postoperative day 4. For maintenance immunosuppression, patients received either tacrolimus (TAC) or cyclosporine (CsA). The starting dose of TAC was 0.15 mg/kg/d, and that of CsA was 5 mg/kg/d. Dosages were adjusted based on therapeutic drug monitoring results. Before the serum creatinine level started to decline, the target trough level of TAC was 4–6 ng/ml, and the target trough level of CsA was 100–150 ng/ml. As allograft function gradually recovered, the trough levels of TAC and CsA were adjusted based on our routine protocol. The target trough level of TAC was 6-8 ng/ml during weeks 1–4, and 5–7 ng/ml thereafter. The target trough level of CsA was 150–200 ng/ml during weeks 1–4, 120–180 ng/ml during month 1–3, and 100–150 ng/ml thereafter.

Patients received either mycophenolate mofetil at a dose of 1–1.5 g/d, or enteric-coated mycophenolate sodium at a dose of 1.08–1.44 g/d. All patients received methylprednisolone at a dose of 5–10 mg/kg/d during the transplant operation, and then daily for the next 2 days. On postoperative day 3, patients were begun on oral prednisone (30 mg/day), and quickly tapered to a maintenance dose of 2.5–10 mg/day by 2 weeks after transplantation.

### Patient follow-up and data collection

After discharge, postoperative follow-up was conducted every week in the first three months and every one to two weeks from month 3 to month 6, every two to four weeks from month 6 to month 12, and every 1–3 months thereafter. At each follow-up visit, the patient’s body weight, vital signs, and immunosuppressive regimen and drug dosages were recorded. Blood was collected for biochemical testing, and for measurement of TAC or CsA levels. Urinalysis was also performed. Glomerular filtration rate was estimated (eGFR) using the Modification of Diet in Renal Disease Study (MDRD) formula for recipients >16 years old, or the Schwartz formula for recipients ≤16 years old. The presence of complications, such as acute rejection or infection, was recorded. If the serum creatinine increased by more than 20% within 72 h, acute rejection was suspected and allograft ultrasonography or biopsy was performed. Biopsy proven acute rejection (BPAR) was based on histopathological examination of the tissue specimen according to Banff Kidney Rejection Classification [26,27]. If a biopsy was not performed, acute rejection was diagnosed after excluding other causes of allograft dysfunction.

### Statistical analysis

Continuous data with normal distribution were presented as mean ± standard deviation, and compared using the t-test (homogeneity of variance), or corrected *t*-test **(**heterogeneity of variance). Continuous data without a normal distribution were expressed as median (interquartile range [IQR]), and compared using the Wilcoxon rank-sum test. Categorical data were reported as counts and percentages, and compared using the chi-square or Fisher’s exact test, as appropriate. Ranked data were presented as median (IQR) and compared using the Wilcoxon rank sum test. Survival analysis was conducted using the Kaplan–Meier method, and the log-rank test was used to compare two survival curves. Univariate and multivariate Cox proportional hazards regression analyses were used for examination of risk factors for the composite endpoint (summation of acute rejection, pneumonia, graft failure, and death). Variables with a value of *p* < 0.2 in the univariable analysis were considered statistically significant and included in the multivariable analysis. Results were reported as hazard ratio (HR) and 95% confidence interval (CI). Statistical analyses were performed using IBM SPSS statistical software version 22.0 (IBM Corporation, New York, USA). A value of *p* < 0.05 was considered to indicate statistical significance.

## Results

### Demographic and clinical characteristics

A total of 713 consecutive patients received solitary renal transplantation from deceased donors at the First Affiliated Hospital, Sun Yat-sen University between February 2007 and August 2016. Two patients without the record of DGF status, 2 patients with primary non-function (PNF) and 4 patients with early graft loss were excluded. In the remaining 705 recipients, there were 91 recipients with DGF and routine follow-up (12.9%), who were included in the analysis.

The induction regimen for 88 of the DGF patients (96.7%) was ATG, and for 3 patients (3.3%) was basiliximab. For maintenance immunosuppression, 89 patients (97.8%) received TAC and 2 (2.2%) received CsA. Of the 91 patients, 59 (64.8%) received mycophenolate mofetil and 32 patients (35.2%) received enteric-coated mycophenolate sodium.

The median follow-up time was 2.6 years (IQR: 1.4–3.6 years). The median DGF recovery time was 27 days (IQR: 15–45 days). Patients were divided into a prolonged recovery group (≥27 days; *n* = 46), and a rapid recovery group (<27 days; *n* = 45). The recovery time in the prolonged recovery group (44.5 days [IQR: 36.0–56.0 days]) was significantly greater than that of the rapid recovery group (15.0 days [IQR: 7.0–19.0 days]) (*p* < 0.001).

Demographic and pre-transplantation characteristics of donors and recipients are summarized in Table 1. Donors of recipients in the prolonged recovery group had significantly higher terminal serum creatinine than those of recipients in the rapid recovery group. In the prolonged recovery group, the TND after transplantation exceeded 14 days in 15 of the 46 (32.6%) recipients. Trough concentrations of TAC at different time points are shown in Table 2. The TAC trough concentration of the prolonged recovery group was lower than that of the rapid recovery group.

**Table 1.** Demographic and clinical characteristics of donors and recipients at time of transplantation.

	Rapid recovery (*n* = 45)	Prolonged recovery (*n* = 46)	*p-*value^a^
Recipients			
Age (years)	38.0 (30.0–45.0)	31.5 (26.0–42.0)	0.1250
Weight (kg)	56.0 (50.0–62.0)	59.5 (46.5–66.0)	0.4287
Gender (%male)	26 (57.8)	35 (76.1)	0.0632
Secondary transplantation (%)	1 (2.2)	0 (0)	0.4945
History of diabetes (%)	4 (8.9)	3 (6.5)	0.9758
History of blood transfusion (%)	8 (17.8)	10 (21.7)	0.6353
Preoperative PRA positive (%)	3 (6.7)	4 (8.7)	1.0000
Dialysis time (days)	381 (210–740)	357 (147–735)	0.3930
HLA mismatch	4 (4–4)	4 (4–4)	0.8825
TND (<14 days/≥14 days)	45/0	31/15	0.0001
TND (days)	1 (1–4)	9 (2–15)	<0.0001
Induction (ATG/basiliximab)	43/2	45/1	0.6166
Calcineurin inhibitor (tacrolimus/cyclosporine)	43/2	46/0	0.2418
Antiproliferative agents (mycophenolate mofetil/mycophenolate sodium)	28/17	31/15	0.6641
Donors			
Age (years)	30.0 (16.0–41.0)	32.0 (19.0–41.0)	0.6640
Weight (kg)	60.0 (32.5–68.0)	60.0 (45.0–70.0)	0.7863
Warm ischemia time (mins)	3.0 (0–10.0)	5.0 (0–15.0)	0.3213
Cold ischemia time (hours)	10.5 (8.0–24.0)	13.5 (10.7–24.0)	0.1282
Terminal serum creatinine (μmol/L)	73.0 (55.5–191.5)	226.5 (149.0–331.0)	0.0049
Terminal eGFR (ml/min/1.73m^2^)	65.4 (36.1–148.9)	24.9 (20.0–52.2)	0.0036
History of hypertension (%)	7 (15.6)	6 (13.0)	0.7321
Cardiac death donors (%)	29 (62.2)	31 (69.6)	0.4599
Cause of death – hypoxia (%)	0 (0)	2 (4.4)	0.4843
Cause of death – cerebrovascular accident (%)	6 (13.3)	5 (10.9)	0.7185
Extended standard donors (%)	3 (6.7)	3 (6.5)	1.0000

^a^Significant at a level of 0.05.

PRA: panel reactive antibodies; HLA: human leukocyte antigen; TND: time needed on dialysis after kidney transplantation; eGFR: estimated glomerular filtration rate.

**Table 2.** Tacrolimus trough concentrations at different time points.

Time point (postoperative day)	Rapid recovery (*n* = 43)^a^	Prolonged recovery (*n* = 46)	*p-*value
3	5.2 (3.3–9.1)	5.4 (2.6–8.4)	1.0000
7	6.4 (5.3–8.7)	4.9 (3.6–6.1)	0.0016
14	6.5 (5.2–8.2)	5.5 (4.4–8.4)	0.1475
30	7.0 (5.7–8.9)	7.9 (5.9–9.5)	0.4901

^a^Two recipients were removed in this table due to the administration with cyclosporine.

In 81 recipients (89.0%), no cause of DGF could be identified. In the other 10 patients, DGF was caused by a perirenal effusion in 3 patients (3.3%), acute rejection in 1 patient (1.1%), ureteral fistula in 1 patient, lymphatic fistula in 1 patient, renal artery stenosis in 1 patient, renal vein stenosis with ureteral obstruction in 1 patient, renal hematoma combined with urinary fistula in 1 patient, and a wound infection in 1 patient.

### Kidney allograft function

At 1 year after transplantation, the eGFR in prolonged recovery group was significantly lower than that of the rapid recovery group (50.6 ± 20.0 vs. 63.5 ± 21.4 mL/min/1.73 m^2^, *p* = 0.005). The trend remained at 2 years and 3 years posttransplant (2 years: 52.6 ± 22.3 vs. 62.4 ± 19.6 mL/min/1.73 m^2^, *p* = 0.045; 3 years: 50.9 ± 21.4 vs. 61.4 ± 19.1 mL/min/1.73 m^2^, *p* = 0.037).

### Survival and post-transplant complications

**Figure 2.**
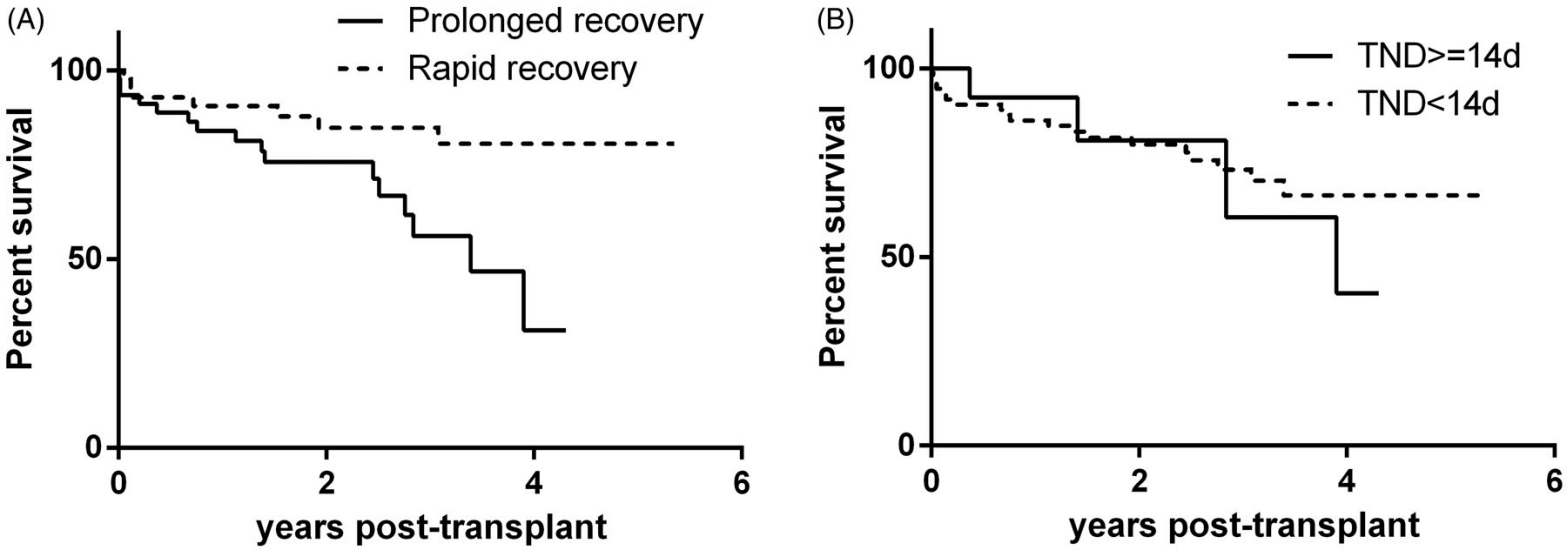
Survival curve free from composite end-point (acute rejection, pneumonia, graft failure and death). (A) Prolonged recovery from DGF (≥27 days) increased the risk of composite end-point (Hazard ratio 2.604, 95% confidence interval 1.102–6.150, *p* = 0.029). DGF recovery time is defined as the time required to achieve stable allograft function from the day of transplantation. (B) TND ≥ 14d was not associated with the increased risk of composite end-point (Hazard ratio 0.869, 95% confidence interval 0.295–2.561, *p* = 0.799). TND, time needed on dialysis after kidney transplantation.

The overall 1-, 2-, and 3-year graft survival rates were 100.0%, 100.0%, and 98.0% respectively, and the 1-, 2-, and 3-year patient survival rates were 100.0%, 100.0%, and 98.0%, respectively. Prolonged recovery from DGF increased the risk of composite end-point (acute rejection, pneumonia, graft failure and death) compared with rapid recovery (Hazard ratio 2.604, 95% confidence interval 1.102–6.150, *p* = 0.029) (Table 3, Figure 2(A)). The 1-year, 2-year and 3-year survival free from composite end-point were 83.9%, 75.7% and 56.0% in prolonged recovery group and 88.5%, 82.9% and 82.9% in rapid recovery group.

**Table 3.** Endpoint events in the prolonged and rapid recovery groups.

Endpoint event	Rapid recovery (*n* = 45)	Prolonged recovery (*n* = 46)	*p-*value
Acute rejection	4 (8.9)	7 (15.2)	0.522
T-cell-mediated	0	4 (8.7)	
Antibody-mediated	0	1 (2.2)	
Clinically-diagnosed	4 (8.9)	2 (4.3)	
Infection (at least one episode)	12 (26.7)	13 (28.3)	0.865
Pneumonia	4 (8.9)	9 (19.6)	
CMV infection	0	2 (4.4)	
Tuberculosis	2 (4.4)	0	
Other	2 (4.4)	7 (15.2)	
Urinary tract infection	5 (11.1)	1 (2.2)	
Gastrointestinal infection	1 (2.2)	1 (2.2)	
Wound infection	1 (2.2)	0	
Other infection	1 (2.2)	2 (4.4)	
IgA nephropathy recurrence	0	1 (2.2)	1.000
FSGS recurrence	0	1 (2.2)	1.000
Malignancy	0	2 (4.4)	0.495
Graft failure	0	1 (2.2)	1.000
Death	0	1 (2.2)	1.000

Data presented as number (percentage).

### Effect of TND on allograft outcomes

The association of time needed on dialysis (TND) after kidney transplantation with the allograft outcome was investigated. Two recent medium-scale studies demonstrated that TND exceeding 14 days was a good indicator of poor allograft function at 1 year [17,18]. Thus, recipients were categorized into 2 groups using 14 days as the cutoff value: <14 days, *n* = 76; ≥14 days, *n* = 15. No significant difference of eGFR at 1 year after transplant was identified between the 2 groups (57.9 ± 21.6 vs. 51.7 ± 21.5 mL/min/1.73 m^2^, *p* = 0.353). A TND ≥14 d was not associated with an increased risk of the composite endpoint (HR = 0.869, 95% CI: 0.295–2.561, *p* = 0.799) (Figure 2(B)).

## Discussion

In this study, DGF recovery time was defined as the time required to achieve stable allograft function as measured from the day of transplantation, which is different than the definition used in prior studies [16–23]. Using this definition, we found that prolonged DGF recovery was associated with poorer 1-year allograft function and higher risk of adverse outcomes (acute rejection, pneumonia, graft failure, and death). We also investigated the effect of TND on allograft outcomes, and found that TND was not an indicator of prognosis. However, this finding may be due to the small number of recipients in the study with a TND ≥ 14 days. In this study, a large proportion of the recipients stopped dialysis early, but recovered slowly after kidney transplantation (e.g. patient 2 in Figure 1). Therefore, DGF recovery time using the definition we propose is likely to be a more sensitive index for prognosis.

We are not the first to realize that the traditional definition of DGF duration has limitations. In 1998, Giral-Classe et al. [22] realized that some patients had to be dialyzed after surgery despite immediate function of the graft because of water and electrolyte imbalances, while in some recipients it was possible to avoid dialysis because clinical and laboratory parameters remained stable after surgery even though they had very low graft function. The authors defined DGF duration as the time required for the kidney to reach a Cockcroft calculated creatinine clearance (cCCr) ≥10 mL/min, and reported that a DGF duration of >6 days was associated with worse graft survival. We did not use that definition in our study because we hypothesized that the evolution of graft function after reaching a cCCr of >10 mL/min remains related to overall prognosis.

We used a composite endpoint as a measure of prognosis in this study. There is evidence that increased TND is associated with increased risk of acute rejection [17,19] and infection [16]. Acute rejection increases the risk of graft loss, and pneumonia after kidney transplantation can be fatal. These complications also adversely affect quality of life, and remarkably increase the overall transplantation cost. Therefore, we believe that using a composite endpoint is superior for evaluating the clinical effect of DGF, as well as examining the pharmacoeconomics of transplantation.

We found that the donor terminal serum creatinine was higher in the prolonged DGF recovery group, while the donor terminal serum creatinine in the rapid recovery group was similar to that in non-DGF group shown in our previous publication [28]. It may indicate that higher donor terminal serum creatinine increases the risk of DGF with prolonged recovery instead of DGF with rapid recovery. This finding reflects the association between the severity of acute kidney injury in the donor and DGF recovery time. Retrospective studies identified an association between increased donor final serum creatinine level and an increased risk of DGF [29,30], but failed to identify an association with prolonged DGF duration among recipients with DGF [17]. Dominguez et al. [24] also investigated risk factors of prolonged DGF duration, and did not find an association with donor kidney function. A study of non-heart-beating donor kidneys found no significant differences in donor serum creatinine levels between recipients with immediate kidney function and those with different durations of DGF (≤2, 2–4, and >4 weeks) [21]. In our study, there were no differences in the warm ischemia time (WIT) and cold ischemia time (CIT) between the prolonged and rapid recovery group, probably due to the relatively short period of ischemia (median 10.5–13.5 h) and the small inter-individual variability. In the previous studies that identified an association of CIT with DGF duration, the average CITs were around 34 h [22] and 23 h [17].

We found that the concentration of calcineurin inhibitors (CNIs: TAC, CsA) in the prolonged recovery group was lower than in the rapid recovery group at 1–2 weeks post-transplant. In the management protocol of DGF at our center, the trough concentration of CNIs is kept relatively low early after transplantation (TAC 4–6 ng/ml). The level is not increased to the normal target range until dialysis is discontinued and renal function begins to recover. Although many transplant surgeons use a low-dose CNI regimen in recipients with DGF, it is still unclear if a low CNI concentration can accelerate the recovery of graft function or improve long-term graft function. Several randomized controlled trials with a large sample size have suggested that lowering CNI exposure neither reduced the incidence of DGF nor shortens DGF recovery time [31]. Our results do not appear to support the use of low-dose CNIs in the management of DGF.

## Conclusion

Decreased donor terminal eGFR is a significant risk factor for prolonged recovery from DGF after deceased kidney transplantation. Prolonged recovery time is associated with decreased graft function at 1 year and poor transplant outcome (acute rejection, pneumonia, graft failure, and death).
